# Partial sleep deprivation after an acute exercise session does not augment hepcidin levels the following day

**DOI:** 10.14814/phy2.14450

**Published:** 2020-05-26

**Authors:** Kazushige Goto, Aoi Mamiya, Hiroto Ito, Tatsuhiro Maruyama, Nanako Hayashi, Claire E. Badenhorst

**Affiliations:** ^1^ Graduate School of Sport and Health Science Ritsumeikan University Shiga Japan; ^2^ School of Sport, Exercise and Nutrition Massey University Auckland New Zealand

**Keywords:** hepcidin, interleukin‐6, iron metabolism, sleep deprivation

## Abstract

The purpose of the present study was to determine the effects of partial sleep deprivation (PSD) after an exercise session in the evening on the endurance exercise‐induced hepcidin response the following morning. Ten recreationally trained males participated under two different conditions. Each condition consisted of 2 consecutive days of training (days 1 and 2). On day 1, participants ran for 60 min at 75% of maximal oxygen uptake ( $\dot{V}$O_2max_) followed by 100 drop jumps. Sleep duration at night was manipulated, with a normal length of sleep (CON condition, 23:00–07:00 hr) or a shortened length of sleep (PSD condition). On the morning of day 2, the participants ran for 60 min at 65% of $\dot{V}$O_2max_. Sleep duration was significantly shorter under the PSD condition (141.2 ± 13.3 min) than under the CON condition (469.0 ± 2.3 min, *p* < .0001). Serum hepcidin, plasma interleukin (IL)‐6, serum haptoglobin, iron, and myoglobin levels did not differ significantly between the conditions (*p* > .05) on the morning (before exercise) of day 2. Additionally, the 3‐hr postexercise levels for the hematological variables were not significantly different between the two conditions (*p* > .05). In conclusion, the present study demonstrated that a single night of PSD after an exercise session in the evening did not affect baseline serum hepcidin level the following morning. Moreover, a 60 min run the following morning increased serum hepcidin and plasma IL‐6 levels significantly, but the exercise‐induced elevations were not affected by PSD.

## Graphical abstract

Endurance exercise increased serum hepcidin levels, but the exercise‐induced elevation was not affected by sleep deprivation.

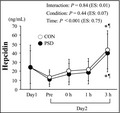

## 1 INTRODUCTION

Iron deficiency is the most common nutrient disorder in the world and is estimated to affect ~50% of the global population (McClung et al., 2009). Iron deficiency or anemia are also frequently observed among endurance athletes (Ishibashi, Maeda, Sumi, & Goto, 2017; Ma, Patterson, Gieschen, & Bodary, 2013). Exercise‐induced iron deficiency is traditionally believed to be related to several factors, including sweating, hemolysis, hematuria, and gastrointestinal bleeding (Zoller & Vogel, 2004). However, attention to hepcidin (an iron regulating hormone) has been growing as a key factor contributing to iron deficiency in endurance athletes (Badenhorst et al., 2016; Peeling et al., 2017). Sufficient evidence has revealed that various types of exercise increase the serum hepcidin level during the postexercise period (Badenhorst et al., 2015a; Domínguez et al., 2018; Goto, Kasai, Kojima, & Ishibashi, 2018; Goto, Sumi, Kojima, & Ishibashi, 2017; Peeling et al., 2017). Moreover, the increase in postexercise hepcidin level has been attributed to exercise‐induced increases in interleukin (IL)‐6 in iron sufficient individuals (Badenhorst et al., 2015a, 2015b; Peeling, 2010). As a support of this idea, lipopolysaccharide (i.e., a protagonist of the inflammatory response) injection increased IL‐6 levels with subsequent elevation of urinary hepcidin levels peaked 3 hr thereafter (Kemna, Pickkers, Nemeth, van der Hoeven, & Swinkels, 2005).

IL‐6 is further facilitated when muscle glycogen levels are lowered (Steensberg et al., 2001). For example, two repeated bouts of endurance exercise on the same day augment the increase in IL‐6 following the second bout of exercise due to lower muscle glycogen levels at the onset of exercise (Ronsen, Lea, Bahr, & Pedersen, 2002). By contrast, the exercise‐induced increase in IL‐6 following a 3 hr run was attenuated by consuming a 6% carbohydrate beverage compared to a placebo beverage (Nieman et al., 1998). In addition to exercise and nutritional status, it appears that sleep deprivation augments IL‐6 production. Skein, Duffield, Edge, Short, and Mündel (2011) determined the effects of a single night of total sleep deprivation during 2 consecutive days of training on muscle glycogen content and found that muscle glycogen content was significantly lower following total sleep deprivation. The authors suggested that the lower muscle glycogen content following total sleep deprivation may be explained by the additional energy expenditure during the night, as energy expenditure is higher during waking than during sleep (White, Weil, & Zwillich, 1985). Moreover, partial sleep deprivation (PSD) has been shown to augment the increase in postexercise IL‐6 levels (Abedelmalek et al., 2013). A recent study by Cullen, Thomas, and Wadley (2020) also demonstrated that a single night of PSD increased the score of subjective fatigue. The author suggested that increased perceptual stress may be involved in elevated IL‐6 following PSD. Additionally, a single night of PSD caused hepatic insulin resistance (Donga et al., 2010). Considering that hepcidin is secreted from liver, disturbance of glucose metabolism in liver might affect hepcidin production.

Although the importance of sleep in athletes is commonly accepted for recovery, athletes frequently experience insufficient sleep (e.g., shortened sleep duration or impaired sleep quality) due to intensive training or night competitions (Kölling et al., 2016; Oda & Shirakawa, 2014; Roberts, Teo, & Warmington, 2019; Sargent, Halson, & Roach, 2014), excessive tension (Fullagar et al., 2016), or during travel for competitions (Bishop, 2004; Eagles, Mclellan, & Hing, 2016; Roberts, Teo, & Warmington, 2019). Even PSD appears to affect exercise performance, and several studies have presented impaired endurance capacity (Chase et al., 2017; Mejri et al., 2016). However, to our knowledge, no study has addressed the effects of PSD on markers of iron metabolism before or following an exercise session completed the morning after PSD.

Thus, we sought to determine the effects of PSD following a strenuous evening exercise session on the endurance exercise‐induced hepcidin response the following morning. We hypothesized that PSD would augment the increase in exercise‐induced IL‐6 and hepcidin the following day.

## 2 MATERIALS AND METHODS

### 2.1 Ethical approval

All participants were informed of the purpose of the study, the experimental procedures, and the possible risks involved in the study, and written informed consent was obtained. They were physically active, but exclusion criteria were having sleep disorders and smoking habit. The present study was approved by the Ethical Committee for Human Experiments at Ritsumeikan University, in accordance with the Declaration of Helsinki.

### 2.2 Participants

Ten recreationally trained male participants (mean ± standard deviation [*SD*], age: 21.6 ± 1.1 years, height: 170.8 ± 5.0 cm, body weight: 62.9 ± 6.3 kg, $\dot{V}$O_2max_: 56.4 ± 3.2 ml kg^−1^ min^−1^) completed the present study. The participants had not been regularly involved in competition at the onset of the present study, but they had exercise habits a few days/week (e.g., resistance exercise, endurance exercise). None were classified as anemic or iron deficient prior to or during the exercise trials.

### 2.3 Experimental overview

Habitual sleep duration was monitored individually over the course of 7 days before the main experiments using actigraphy (GT3XBT; Ambulatory Monitoring Inc.). Moreover, a preliminary session was performed to allow the participants to become familiarized with the exercise, the laboratory environment and to determine maximal oxygen uptake ( $\dot{V}$O_2max_). The main experiments (consisting of two conditions) were started approximately 1 week following the preliminary session. All participants were exposed to the two conditions (CON and PSD) in a crossover design, and the order of each trial was randomized. There was a minimum of 3 weeks between each of the trials. They were also required to go to bed before 24:00 hr in the night prior to each trial.

**FIGURE 1:**
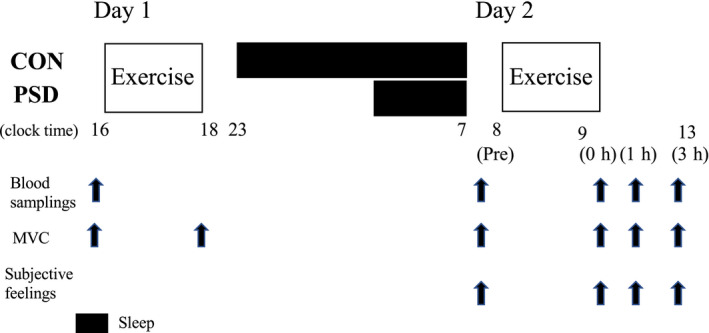
Experimental design. MVC; Evaluation of maximal voluntary contraction

On day 1 of the experiment, the participants ran for 60 min at 75% of $\dot{V}$O_2max_. After 10 min rest, they conducted 100 drop jumps from 60 cm height. Sleep duration at night was manipulated, with a normal length of sleep (CON condition) or a shortened length of sleep (PSD condition). The participants ran for 60 min at 65% of $\dot{V}$O_2max_ the following morning (day 2). Hematological markers of iron status were determined before and following exercise on day 2 and results were compared between trials (Figure 1).

### 2.4 Manipulation of sleep duration (CON and PSD conditions)

Daily sleep duration was recorded for 7 consecutive days before the main experiments using an accelerometer (Actigraph; Ambulatory Monitoring Inc.), and the magnitude of sleep deprivation was determined individually. In the CON condition, the participants were allowed to sleep from 23:00 to 07:00 hr (8 hr). However, in the PSD condition, sleep duration was shortened to match the equivalent of 40% of the individual regular sleep duration (60% reduction from daily sleep duration recorded for 7 consecutive days before the main experiments). The level of PSD was determined based on the previous study which demonstrated that a single night of about 66% of sleep restriction (sleep for about 2.4 hr) following heavy exercise impaired performance during 3 km time trial (Chase et al., 2017). We also utilized PSD during the early phase of the night, because delayed onset of sleep appears to be more realistic among athletes compared with PSD during late phase of the night.

The participants in the PSD condition were requested to stay awake from 23:00 hr to an individually determined onset of sleep to achieve 60% reduction from regular sleep duration (i.e., deprivation of sleep during the early phase of the night). The participants under both conditions woke up at the same time of day (07:00 hr). Participants were encouraged to spend time watching television, reading books, or engaged in other nonexercise activities during the period of sleep deprivation.

### 2.5 Exercise protocols on day 1 and day 2

The participants arrived in the laboratory before 15:00 hr on day 1. They initially received evaluations of blood variables, maximal muscle voluntary contraction (MVC), and muscle soreness score. Subsequently, the participants started a 60 min run at 75% of $\dot{V}$O_2max_ using a treadmill (E95Ta; Life Fitness). After 10 min, they performed 100 drop jumps (10 × 10 sets) from a 60 cm box. Each jump was repeated every 10 s, and 30 s rest was allowed between sets. After landing, the participants were required to fully bend the knee joint (approximately 90°) and the hip joint to cause eccentric muscle contraction of the quadriceps femoris muscles (Jakeman, Byrne, & Eston, 2010). The exercise duration in total (including the 60 min run, 100 drop jumps, rest periods between exercises) was approximately 90 min. The exercise protocol consisting of a 60 min run followed by 100 drop jumps was determined to promote muscle glycogen utilization and exercise‐induced muscle damage to mimic the training situation on team sports (i.e., soccer, rugby).

Prescribed meals were provided on day 1 (lunch: carbohydrate 65%, protein 15% fat 20%, 854 kcal; carbohydrate 71%, protein 10% fat 19%907 kcal) for each condition.

The participants returned to the laboratory on day 2 (08:00 hr), following an overnight fast (at least 12 hr following previous meal). They ran for 60 min on the treadmill at 65% of $\dot{V}$O_2max_. We selected a lower exercise intensity (65% of $\dot{V}$O_2max_) on day 2 due to the possibility that the participants could not complete the same exercise as on day 1 (60 min run at 75% of $\dot{V}$O_2max_) due to muscle damage induced by the drop jumps. Following the completion of the exercise session, participants rested in the laboratory for a further 3‐hr period. On day 2, the participants were not allowed to eat meals until 3 hr after completing exercise.

### 2.6 Measurements

#### 2.6.1 $\dot{V}$O_2max_

An incremental running test was conducted at multiple stages to evaluate $\dot{V}$O_2max_. Initial running velocity was set to 6 km/hr, and running velocity was increased by 0.6–2.0 km/hr every 1–3 min until volitional exhaustion (Sumi, Kojima, & Goto, 2010). During the test, expired gases were collected and analyzed using an automatic gas analyzer (AE300S; Minato Medical Science). The data were averaged every 30 s. Heart rate was measured continuously during the test using a wireless heart rate monitor (Accurex Plus; Polar Electro Oy).

#### 2.6.2 Sleep quality

Sleep quality was evaluated using a mattress sensor during the night of day 1 (Sleep Scan SL‐511‐WF‐2; Tanita). Data collection lasted from the onset of sleep to waking on day 2. Total sleep time, sleep onset latency, and sleep efficiency were evaluated for each trial.

#### 2.6.3 Blood variables

Blood samples were collected from an antecubital vein five times under each condition: before exercise on days 1 and 2, immediately after exercise, and 1 and 3 hr after exercise on day 2 (Figure 1). After blood collection, serum and plasma samples were obtained by centrifugation (10 min, 3,000 rpm, 4°C) and stored at −80°C until further analysis. Blood glucose, lactate, iron metabolism‐related markers (serum hepcidin, iron levels), indirect muscle damage and inflammatory markers (myoglobin [Mb], creatine kinase [CK], high‐sensitive C reactive protein [hs‐CRP], and plasma IL‐6 levels) were evaluated. Serum iron, Mb, CK, and hs‐CRP levels were measured at a clinical laboratory (SRL). The intra‐assay coefficients of variation (CVs) for these assays were 1.7% for serum iron, 2.9% for Mb, 2.0% for CK, and 1.5% for hs‐CRP. Serum hepcidin and plasma IL‐6 levels were analyzed by enzyme‐linked immunosorbent assays (ELISA) using commercially available kits (R&D Systems) (Goto et al., 2018; Ishibashi et al., 2017). The intra‐assay CVs for the hepcidin and IL‐6 assays were 1.7% and 2.5%, respectively. Blood glucose and lactate levels were measured immediately after blood collection using a glucose analyzer (Freestyle, Nipro Corp.) and a lactate analyzer (Lactate Pro, Arkray, Inc.), respectively.

#### 2.6.4 Maximal muscle strength

MVC for knee extension was evaluated before exercise on day 1, before exercise on day 2, immediately after exercise, and 1 and 3 hr after exercise on day 2 using an isokinetic dynamometer (Biodex System 4; SAKAI Medical Co.). MVC was measured at a knee angle of 110° (full extension of the leg was defined as 180°), and participants were requested to exert maximal strength for 3 s. Each measurement was repeated twice, and the highest value was selected for further analysis.

#### 2.6.5 Muscle soreness score

The subjective muscle soreness score was assessed using a 100‐mm visual analog scale before exercise, immediately after exercise, and 3 and 24 hr after exercise. The 0‐mm mark indicated no pain, and the 100‐mm mark indicated the worst pain imaginable (Roberts, Nosaka, Coombes, & Peake, 2014).

### 2.7 Statistical analysis

All data are presented as mean ± *SD*. Time‐course changes in the blood variables, MVC, and muscle soreness scores were compared using two‐way repeated‐measures analysis of variance to confirm the interaction (condition × time) and the main effects. Where a significant interaction or main effect was evident, a post‐hoc Tukey–Kramer test was performed to detect differences. To reveal the magnitude of difference, effect size (ES) was calculated using partial *η* ^2^. Variables for sleep quality on day 1 were evaluated by the paired *t*‐test. A *p*‐value <.05 was considered significant.

## 3 RESULTS

### 3.1 Sleep quality and subjective feeling scores

During the night on day 1, sleep quality was significantly different between the two conditions (evaluated using a mattress sensor). Total sleep time was significantly shorter under the PSD condition (141.2 ± 13.3 min) than under the CON condition (469.0 ± 2.3 min, *p *< .0001). Sleep latency did not differ significantly between the PSD condition (3.1 ± 3.4 min) and the CON condition (8.1 ± 5.9 min, *p* = .28), whereas sleep efficiency was significantly higher under the PSD condition (93.4 ± 3.1%) than under the CON condition (87.6 ± 2.4%*, p *= .02).

Before exercise on day 2, the sleepiness score was significantly higher under the PSD condition than under the CON condition (condition: *p *= .04). Exercise significantly decreased the sleepiness score under the PSD condition, but the scores postexercise on day 2 were still significantly higher under the PSD condition (condition × time: *p* = .005, time: *p *= .001). The fatigue score was significantly higher under the PSD condition on the morning of day 2 prior to the exercise session (condition × time: *p *= .005, condition: *p *= .007). However, no significant difference between conditions was observed during the postexercise period. Muscle soreness did not differ significantly between conditions (condition × time: *p *= .61, condition: *p *= .21, Table 1).

**TABLE 1:** Scores of sleepiness, fatigue and muscle soreness

		Pre	0 hr	1 hr	3 hr	Interaction	Condition	Time
Sleepiness (mm)	CON	38 ± 27	13 ± 11	37 ± 27	38 ± 31	*p* = .002	*p* = .042	*p* = .001
	PSD	83 ± 13^†^	18 ± 22*	44 ± 31*	42 ± 31*	(0.41)	(0.38)	(0.52)
Fatigue (mm)	CON	46 ± 19	71 ± 13*	53 ± 16	47 ± 22	*p* = .236	*p* = .070	*p* = .015
	PSD	67 ± 12	77 ± 13	57 ± 19	56 ± 27	(0.14)	(0.32)	(0.44)
Muscle soreness (mm)	CON	51 ± 16	64 ± 16	67 ± 18*	68 ± 17*	*p* = .647	*p* = .212	*p* = .033
	PSD	64 ± 19	71 ± 13	73 ± 16	71 ± 22	(0.06)	(0.17)	(0.27)

Note

Values are means ± *SD*.

* *p* < .05 versus Pre.

^†^ *p* < .01 between conditions.

### 3.2 Hepcidin

**FIGURE 2:**
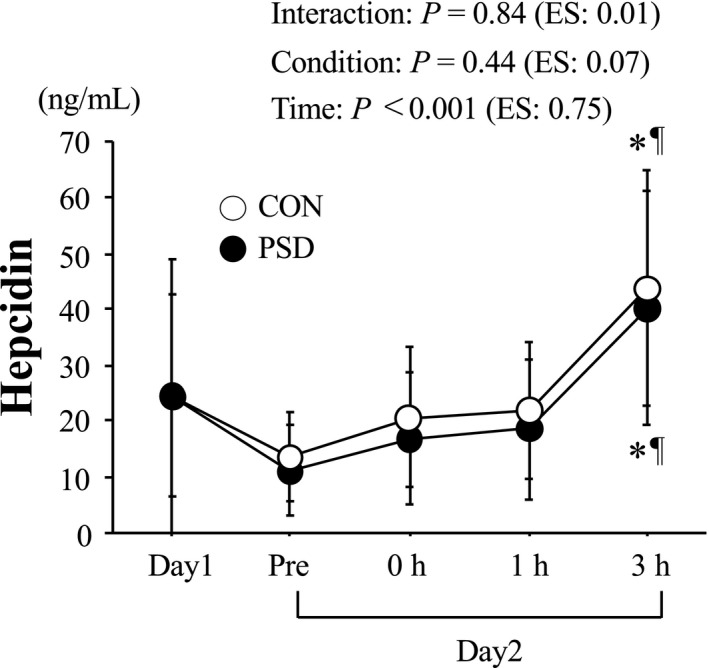
Change in serum hepcidin level. Values are means ± *SD*. *p < .05 versus Day 1. ^¶^ *p* < .05 versus Pre. Effect size (ES) was calculated using partial *η* ^2^

The changes in serum hepcidin levels are shown in Figure 2. No significant difference in serum hepcidin level was observed between the conditions on day 1. On day 2, serum hepcidin levels increased significantly after exercise under both conditions (time: *p* < .001). However, no significant difference was observed at 3‐hr postexercise (condition × time: *p* = .99, condition: *p* = .44).

### 3.3 Other blood variables

**FIGURE 3:**
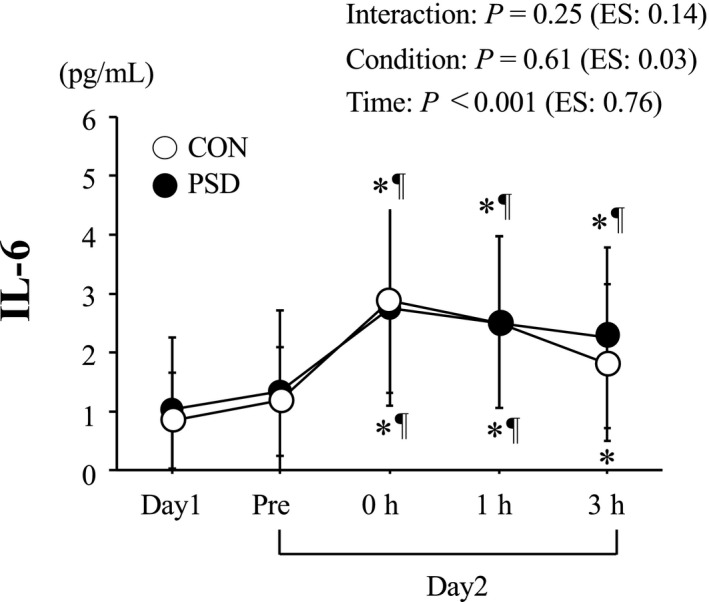
Plasma IL‐6 level. Values are means ± *SD*. *p < .05 versus Day 1. ^¶^ *p* < .05 versus Pre. Effect size (ES) was calculated using partial *η* ^2^

The changes in plasma IL‐6 levels are shown in Figure 3. No significant difference in plasma IL‐6 level was observed between the conditions on day 1. On day 2, plasma IL‐6 levels increased significantly after exercise for both conditions (time: *p* < .001). However, no significant difference was observed at 3‐hr postexercise (condition × time: *p* = .25, condition: *p *= .61).

On day 2, serum haptoglobin levels decreased significantly after exercise for both conditions (time: *p* < .001), but no significant difference was observed between conditions (condition × time: *p* = .58, condition: *p *= .78). By contrast, serum iron levels increased significantly after exercise for both conditions (time: *p* < .001). However, serum iron levels during the 3‐hr postexercise time period were not significantly different between conditions (condition × time: *p* = .38, condition: *p* = .96, Table 2).

**TABLE 2:** Serum haptoglobin and iron levels

		Day 1	Day 2						
			Pre	0 hr	1 hr	3 hr	Interaction	Condition	Time
Haptoglobin (mg/dl)	CON	45.3 ± 28.6	34.0 ± 31.4*	32.5 ± 31.7*	29.3 ± 28.9*	28.1 ± 27.4*	*p* = .439	*p* = .777	*p* < .001
	PSD	45.3 ± 44.4	33.0 ± 36.7*	30.2 ± 35.4*	23.8 ± 26.0*	28.3 ± 35.3*	(0.08)	(0.01)	(0.75)
Iron (μg/dl)	CON	145 ± 49	176 ± 40*	215 ± 40*, †	206 ± 33*	207 ± 33*, †	*p* = .337	*p* = .959	*p* < .001
	PSD	125 ± 33	167 ± 53*	209 ± 63^*^, ^†^	212 ± 54^*^, ^†^	225 ± 51^*^, ^†^	(0.11)	(<0.01)	(0.88)

Note

Values are means ± *SD*.

* *p* < .05 versus Day1.

^†^ *p* < .05 versus Pre.

Serum Mb and CK levels were significantly higher before exercise on day 2 than those on day 1. Serum Mb and CK levels further increased after exercise on day 2 (time: *p *< .001 for both variables), but no difference was observed between conditions at 3‐hr postexercise time period (Mb; condition × time: *p *= .20, condition: *p *= .67, CK; condition × time: *p *= .84, condition: *p *= .90). Serum hs‐CRP levels increased significantly on day 2 (vs. day 1) but only for the CON condition (time: *p* = .002). However, no significant difference between conditions was observed at any time points on day 2 (condition × time: *p *= .30, condition: *p *= .77, Table 3).

**TABLE 3:** Serum myoglobin (Mb), creatine kinase (CK) and high‐sensitive C reactive protein (hs‐CRP) levels

		Day 1	Day 2						
			Pre	0 hr	1 hr	3 hr	Interaction	Condition	Time
Mb (ng/ml)	CON	23.7 ± 4.6	58.1 ± 17.2	182.8 ± 86.5^*^, ^†^	220.6 ± 107.1^*^, ^†^	150.8 ± 67.7^*^, ^†^	*p* = .235	*p* = .686	*p* < .001
	PSD	39.6 ± 32.1	62.0 ± 16.3	172.2 ± 84.5^*^, ^†^	197.9 ± 90.2^*^, ^†^	143.1 ± 56.5^*^, ^†^	(0.15)	(0.02)	(0.79)
CK (U/L)	CON	119 ± 34	312 ± 216*	448 ± 328*	427 ± 300*	441 ± 147*	*p* = .571	*p* = .895	*p* = .001
	PSD	150 ± 55	287 ± 97*, ^†^	425 ± 135^*^, ^†^	415 ± 126^*^, ^†^	334 ± 188^*^, ^†^	(0.04)	(0.002)	(0.74)
hs‐CRP (ng/ml)	CON	134 ± 103	288 ± 150*	321 ± 170*	316 ± 167*	334 ± 188*	*p* = .298	*p* = .765	*p* = .049
	PSD	303 ± 338	347 ± 205	298 ± 190	309 ± 169	317 ± 170	(0.18)	(0.02)	(0.50)

Note

Values are means ± *SD*.

* *p* < .05 versus Day1.

^†^ *p* < .05 versus Pre.

There were no significant differences in blood glucose or lactate levels before exercise on day 2. Blood glucose levels decreased significantly after exercise on day 2 (time: *p *= .01), whereas blood lactate levels increased significantly after exercise (time: *p *= .01). However, the exercise‐induced changes in each variable did not differ significantly between conditions (glucose; condition × time: *p* = .28, condition: *p *= .26, lactate; condition × time: *p *= .35, condition: *p *= .29).

### 3.4 MVC

Table 4 presents the changes in MVC. Exercise on day 1 decreased MVC significantly immediately after exercise in CON condition (*p* < .05). MVC was significantly lower before exercise on day 2 than that on day 1 in both conditions (time: *p *< .001). However, MVC did not decrease after exercise on day 2. Furthermore, no significant difference was observed between conditions at any time point (condition × time: *p* = .56, condition: *p *= .93).

**TABLE 4:** Maximal voluntary contraction (MVC) for knee extension exercise

		Day 1		Day 2						
		Pre	0 hr	Pre	0 hr	1 hr	3 hr	Interaction	Condition	Time
MVC (Nm)	CON	245 ± 35	224 ± 39*	210 ± 43*	203 ± 55*	210 ± 42*	222 ± 50*	*p* = .487	*p* = .929	*p* < .001
	PSD	244 ± 40	228 ± 46	209 ± 47*	206 ± 53*	213 ± 50*	212 ± 51*	(0.08)	(0.01)	(0.68)

Note

Values are means ± *SD*.

* *p* < .05 versus Pre (Day 1).

## 4 DISCUSSION

The novelty of the present study was to focus on the impact of partial sleep deprivation after exercise on exercise‐induced hepcidin elevation the following morning. Although majority of previous studies determined how the reduced sleep duration affected exercise performance the following day, we were aware that no information was available to demonstrate the influences of partial restriction of nocturnal sleep on condition‐related variables (e.g., iron metabolism, muscle damage, and inflammatory markers). The main finding of the present study was that acute PSD after an exercise session in the evening did not augment the increase in endurance exercise‐induced hepcidin the following morning. Moreover, no harmful effects of acute PSD were observed on exercise‐induced muscle damage or the inflammatory response.

In the PSD condition, sleep duration during the night was shortened 60% from individual normal sleep duration, which was recorded over 7 days prior to the main experiment. We preferentially chose PSD condition instead of total sleep deprivation on day 1, as PSD is considered a more realistic condition that can occur among the athletes and the general population (Kölling et al., 2016; Oda & Shirakawa, 2014; Sargent et al., 2014). We can conclude that our PSD condition was effective as results demonstrated significantly shorter sleep duration, with augmented sleepiness before exercise on day 2.

Serum hepcidin levels did not differ significantly before exercise on day 2 between the CON and PSD conditions. Although 60 min of running at 65% of $\dot{V}$O_2max_ on day 2 increased the serum hepcidin level significantly 3 hr after the exercise, the magnitude of the increase in exercise‐induced hepcidin did not differ significantly between the PSD and CON conditions. Several factors could influence the increase in hepcidin following acute endurance exercise, but exercise‐induced IL‐6 production has been thought to be the primary factor (Peeling, 2010; Sim et al., 2012). We had speculated that the exercise‐induced increase in IL‐6 would be augmented under the PSD condition, a result that could be due to a night of sleep deprivation after an exercise session decreasing muscle glycogen content the following morning (Skein et al., 2011). Additional energy expenditure while remaining awake during PSD may be responsible for the lowered muscle glycogen content the following morning. The lower muscle glycogen content at the onset of endurance exercise has been shown to augment IL‐6 production from working muscles (Steensberg et al., 2001). However, we were unable to determine muscle glycogen content the following PSD condition of the current trial. Considering that neither blood glucose level nor fat oxidation on the morning of day 2 differed significantly between the conditions (data not shown), it appears that there was no apparent difference in muscle glycogen content between the conditions on day 2. Therefore, we can suggest that the food (dinner) provided following the exercise session on day 1 was effective in mitigating muscle glycogen depletion even during PSD trial. This may suggest that adequate nutrition provided following an evening session may be effective in attenuating the stimulus (muscle glycogen depletion) for exacerbated IL‐6 in CON and PSD conditions in athletes. In addition to IL‐6, baseline iron levels (Peeling et al., 2017) and the postexercise increase in serum iron resulting from hemolysis (Peeling et al., 2009) are additional factors to consider, that will facilitate increased hepcidin production. However, serum iron and haptoglobin levels (an indication of exercise‐induced hemolysis) before exercise on day 2 or during 3‐hr postexercise did not differ significantly between the conditions. Therefore, the lack of a difference in the postexercise increase in hepcidin on day 2 between the two conditions is understandable.

Exercise on day 1 consisted of 60 min of running at 75% $\dot{V}$O_2max_ followed by 100 drop jumps, and this increased the indirect blood markers of muscle damage (i.e., serum Mb and CK levels) and decreased MVC before exercise on day 2. However, no significant differences in these variables were found between the conditions. Additionally, the increases in Mb, CK, and hs‐CRP following acute endurance exercise on day 2 were not significantly different between conditions, suggesting that PSD after a damaging exercise session on day 1 did not exacerbate recovery of muscle function or the muscle damage the following morning. By contrast, 1–2 days of total sleep deprivation or 10 days of PSD has been shown to increase hs‐CRP level (Meier‐Ewert et al., 2004). Moreover, 3 consecutive days of PSD (sleep deprivation of 3 hr per night) has been shown to reduce maximal muscular strength during bench press, leg press, and deadlift exercises (Reilly & Piercy, 1994). Considering our trials lasted for a single night, it is possible that the severity of sleep deprivation (total sleep deprivation or PSD) or length of sleep loss (a single night of PSD or consecutive days of PSD) may have affected the present outcomes, and future research in long trials (3–7 days) may be warranted.

The present study includes some limitations for the interpretation of the results. Firstly, we were not able to evaluate muscle glycogen content. Unfortunately, no previous studies have been conducted so far to investigate the relations between exercise‐induced muscle glycogen decrement and hepcidin elevation. In our latest study, we have observed that change in muscle glycogen content significantly correlated with the change in serum hepcidin level (unpublished data). Thus, determination of muscle glycogen content would contribute to clarifying the impact of PSD following an endurance exercise session on iron metabolism. Secondly, because we manipulated sleep duration during a single night, the cumulative effects of PSD during a training period remain unclear. Some previous studies that have used consecutive days of PSD have presented attenuated muscle function (Reilly & Piercy, 1994) and endurance capacity (Roberts, Teo, Aisbett, & Warmington, 2019), however, their findings are not fully consistent (Spencer, Bishop, Dawson, & Goodman, 2005). Thirdly, the present study shortened sleep duration by 60% during the early phase of the night. This is because the early phase of the night includes a higher ratio of slow‐wave sleep (stages 3 and 4), which plays an important role in recovery (Abedelmalek et al., 2013). However, in a previous study by Mejri et al. (2017), PSD during the latter phase of the night (0300–0600) caused a greater attenuation of endurance capacity the following morning when compared with PSD during the early phase of the night (22:30–03:00 hr). Additionally, PSD during the latter phase of the night has been shown to augment Mb and hs‐CRP levels (Mejri et al., 2016). Therefore, the influence of different phases of PSD (early phase vs. latter phase) on iron metabolism would be a valuable topic for a future study.

## 5 CONCLUSION

The present study demonstrated that 60% of PSD after an exercise session in the evening did not affect baseline serum hepcidin levels the following morning. Moreover, 60 min of running the following morning increased serum hepcidin and plasma IL‐6 levels significantly, but the exercise‐induced increases were not affected by PSD. These findings suggest that exercise‐induced iron metabolism is relatively robust despite a single night of sleep loss after an exercise session.

## CONFLICT OF INTEREST

The authors declare no conflict of interest and have no financial relationship to disclose.

## AUTHORS’ CONTRIBUTIONS

KG and AM were part of the conception and protocol design, and wrote the manuscript. KG, AM, HI, and TM conducted the experiments. KG, AM, and NH were responsible for data analyses. CEB critically revised the manuscript and gave advices for corrections to KG.
